# Differential protein stability of EGFR mutants determines responsiveness to tyrosine kinase inhibitors

**DOI:** 10.18632/oncotarget.11860

**Published:** 2016-09-06

**Authors:** Paramita Ray, Yee Sun Tan, Vishal Somnay, Ranjit Mehta, Merna Sitto, Aarif Ahsan, Shyam Nyati, John P. Naughton, Alexander Bridges, Lili Zhao, Alnawaz Rehemtulla, Theodore S. Lawrence, Dipankar Ray, Mukesh K. Nyati

**Affiliations:** ^1^ Department of Radiation Oncology, University of Michigan, Ann Arbor, MI 48109, USA; ^2^ School of Pharmacy, University of Michigan, Ann Arbor, MI 48109, USA; ^3^ Department of Biostatistics, University of Michigan, Ann Arbor, MI 48109, USA; ^4^ Current address: Oncology Research Unit East, Pfizer, Pearl River, NY 10965, USA; ^5^ Current address: Department of Otorhinolaryngology-Head and Neck Surgery, Albert Einstein College of Medicine, Montefiore Medical Center, Bronx, NY 10467, USA

**Keywords:** EGFR, erlotinib, protein stability, ubiquitination, TKI sensitivity

## Abstract

Non-small cell lung cancer (NSCLC) patients carrying specific EGFR kinase activating mutations (L858R, delE746-A750) respond well to tyrosine kinase inhibitors (TKIs). However, drug resistance develops within a year. In about 50% of such patients, acquired drug resistance is attributed to the enrichment of a constitutively active point mutation within the EGFR kinase domain (T790M). To date, differential drug-binding and altered ATP affinities by EGFR mutants have been shown to be responsible for differential TKI response. As it has been reported that EGFR stability plays a role in the survival of EGFR driven cancers, we hypothesized that differential TKI-induced receptor degradation between the sensitive L858R and delE746-A750 and the resistant T790M may also play a role in drug responsiveness. To explore this, we have utilized an EGFR-*null* CHO overexpression system as well as NSCLC cell lines expressing various EGFR mutants and determined the effects of erlotinib treatment. We found that erlotinib inhibits EGFR phosphorylation in both TKI sensitive and resistant cells, but the protein half-lives of L858R and delE746-A750 were significantly shorter than L858R/T790M. Third generation EGFR kinase inhibitor (AZD9291) inhibits the growth of L858R/T790M-EGFR driven cells and also induces EGFR degradation. Erlotinib treatment induced polyubiquitination and proteasomal degradation, primarily in a c-CBL-independent manner, in TKI sensitive L858R and delE746-A750 mutants when compared to the L858R/T790M mutant, which correlated with drug sensitivity. These data suggest an additional mechanism of TKI resistance, and we postulate that agents that degrade L858R/T790M-EGFR protein may overcome TKI resistance.

## INTRODUCTION

The epidermal growth factor receptor (EGFR) family of receptor tyrosine kinases (TK) regulates major developmental and metabolic processes. The kinase activity of EGFR is often dysregulated in tumor cells, and its aberrant activation can lead to enhanced cell survival, proliferation, invasion, and metastasis [1]. Activating somatic mutations in EGFR are prevalent in non-small cell lung cancer (NSCLC) patients [2]. There are two activating mutations of the EGFR gene that together constitute about 90% of all EGFR activating mutations: in-frame deletions in exon 19 (delE746-A750) and a point mutation in exon 21 that substitutes an arginine for a leucine at codon 858 (L858R). In addition to these frequent mutations about 5% of lung cancer patient tumors contain an insertion in exon 20 between amino acids 767 to 774 [3]. These activating mutations lead to an equilibrium shift with ATP that favors the activated TK state leading to an increase in kinase activity, and, thus, the tumor cells displaying these mutations have growth and survival advantages [4]. Although the EGFR tyrosine kinase inhibitors (TKI) have shown activity in NSCLC, acquired resistance to these agents ultimately leads to disease progression within a year. In approximately half of these cases, resistance is due to the occurrence of a point mutation in EGFR Exon 20 (T790M) [5]. This threonine at residue 790 is considered the gatekeeper residue which controls ATP recruitment by the kinase domain of EGFR.

Current studies suggest two potential mechanisms by which cells acquire TKI resistance. The first is that the T790M mutant has about 10 to 15 fold higher affinity towards ATP when compared to activating EGFR mutants. Thus, TKIs cannot displace ATP as efficiently as they can in the case of L858R-EGFR where ATP binding is relatively weaker [6]. The second hypothesis is that replacement of threonine by bulkier methionine causes steric hindrance which limits the binding of TKIs with the EGFR kinase domain [7]. However, it remains possible that there are other mechanisms of acquired TKI resistance. For example, with regard to the first mechanism, it is known that the K_m_ values for ATP binding of wild-type and TKI resistant T790M-EGFR are similar [6, 8], but they respond differently to TKI treatment. The second hypothesis, related to limited binding with TKI when threonine is replaced with bulkier methionine, is also attractive, but several biochemical and structural studies suggested that M790 may not sterically impede most “quinazoline” inhibitors from binding [6] (PDB 4LL0). Furthermore, irreversible inhibitors bind to the double mutants (L858R with T790M) with only slightly diminished affinity compared to wild-type enzyme [9], or, with greater affinity as seen with the bulky inhibitor Neratinib, which was found to have a K_i_ 50-fold more potent for L858R/T790M EGFR than for wild-type EGFR [10]. This finding is supported by recent data that show “mutant-selective” irreversible TKIs such as AZD9291 have similar inhibitory potency for L858R/T790M to the first generation of TKI's but reduced affinity for wild-type EGFR [11].

Therefore, although the clinical observation correlating EGFR mutations with the response has long been established, the precise mechanism(s) of sensitivity or resistance to TKI still remain unclear. We and others have noted that EGFR degradation upon gemcitabine, cisplatin or radiation increases tumor cell-specific cytotoxicity beyond that of EGFR inhibition alone [12-19], and TKI-resistant cells are responsive to therapies that induce EGFR degradation [19-22]. Therefore, we hypothesized that differential EGFR degradation may contribute to the difference in sensitivity to TKIs observed in patients harboring L858R as compared to those who co-harbor T790M. EGFR protein degradation upon ligand (EGF) binding is well established, where c-CBL ubiquitin ligase is implicated in phospho-EGFR polyubiquitination and lysosomal degradation. However, the involvement of c-CBL in erlotinib-induced receptor degradation of inactive receptor remain unknown.

To test our hypothesis, we characterized the effect of erlotinib on EGFR protein stability in cells harboring drug sensitive or resistant mutations frequently observed in patients within the kinase domain. For the mechanistic studies, we constructed YFP fusion constructs with the L858R mutation, the L858R/T790M double mutant, and wild-type full-length EGFR (as control). We used EGFR-null Chinese hamster ovary (CHO) cells to overexpress individual EGFR constructs and visualized EGFR expression and monitored changes in their steady state levels and localization upon erlotinib treatment. Additionally, we utilized NSCLC and HNSCC cell lines expressing endogenous mutant EGFR proteins to study the effect of erlotinib on EGFR protein half-lives. The mechanistic role of c-CBL in erlotinib mediated EGFR degradation was also determined. Finally, using a genetically modified sub-line of NCI-H1975 cells, we conducted *in vivo* experiments and imaged EGFR activity in real-time using a non-invasive bioluminescence reporter and also assessed the effect of treatment on tumor growth. In this model, we found that, although erlotinib blocked EGFR activity, tumor growth was not affected. These findings suggest that EGFR protein stability, not just its activity plays an important role in erlotinib response.

## RESULTS

### Erlotinib treatment induces rapid downregulation of L858R-YFP protein following intracellular aggregation in CHO cells

**Figure 1:**
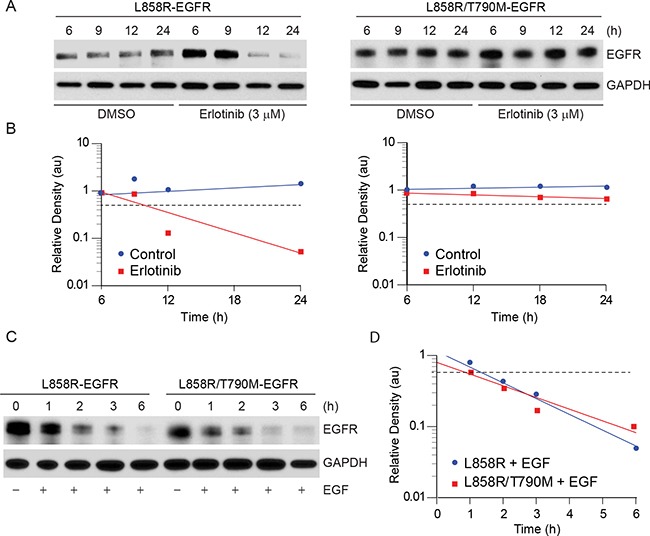
Erlotinib treatment results in faster downregulation of L858R-YFP protein **A.** CHO cells transiently expressing either L858R or L858R/T790M mutant YFP-EGFR were either treated with vehicle (DMSO) control or with 3 μM erlotinib. Cell lysates were prepared at the indicated time points and immunoblotted using the indicated antibodies. **B.** Individual band intensity (arbitrary units, au) was calculated using Image J software, and relative band densities were plotted against time. **C.** Transiently transfected CHO cells expressing either L858R or L858R/T790M mutant YFP-EGFR were either left untreated or treated with 10 ng/ml EGF for the indicted times, and cell lysates were immunoblotted using the indicated antibodies. **D.** Relative band intensities were calculated as described in panel B and plotted with time.

To study the effect of erlotinib on different EGFR mutants, we used a transient transfection system using CHO cells, which do not express endogenous EGFR. We constructed and sequence verified EGFR-YFP constructs including L858R and L858R/T790M mutants using site-directed mutagenesis. Equal amounts of DNA were then individually transfected into CHO cells, and 12 h post-transfection cells were treated either with vehicle (DMSO) or with 3 μM erlotinib. We selected this concentration of erlotinib based on a pharmacodynamic study in humans that showed that the C_max_ of erlotinib is about 3.5 μM [23]. Immunoblotting analyses indicated that erlotinib treatment caused faster decay of L858R mutant protein when compared to L858R/T790M double mutant (Figure 1A, 1B). In contrast, EGF treatment, which downregulates EGFR [24], was found to be equally efficacious in downregulation of both L858R and L858R/T790M mutants (Figure 1C, 1D), suggesting that erlotinib selectively induces EGFR degradation only in the cells that contain activating EGFR mutations. In this model, wild-type (WT) EGFR also showed sensitivity similar to L858R mutant in response to both EGF and erlotinib (Supplementary Figure S1A, S1B). We also assessed the effect of erlotinib on EGFR localization in the live cells using fluorescence microscopy at 2, 8, 18, and 24 h post treatment. YFP-EGFR (L858R) mutant expressing cells showed more cytosolic expression with larger protein aggregates, as opposed to predominantly membranous localization noted in the L858R/T790M mutant cells (Supplementary Figure S2A, upper panel). Furthermore, within 2 h of erlotinib treatment, there was about a 3 fold increase in cytosolic protein aggregation in L858R mutant cells followed by a rapid decay in fluorescence intensity between 8-12 h of drug treatment (Supplementary Figure S2B). These data are consistent with the immunoblotting data as shown in Figure 1A. In contrast, change in localization and fluorescence intensity were minimal for L858R/T790M mutant cells during the observation period of 24 h (Supplementary Figure S2, lower panel).

### Erlotinib treatment induces rapid down-regulation of L858R and delE746-A750 EGFR proteins in lung cancer cells

**Figure 2:**
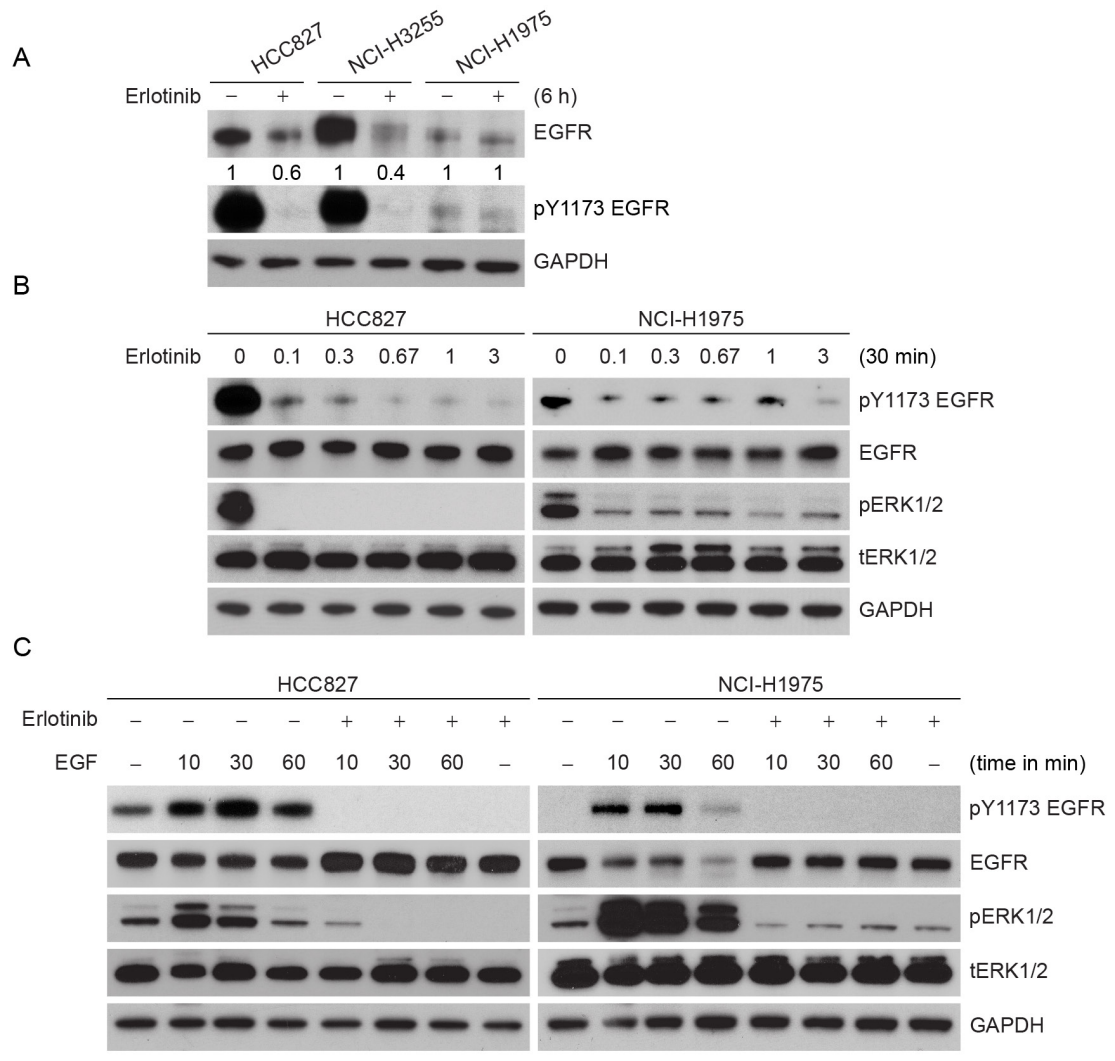
Effect of erlotinib on different lung cancer cells carrying different EGFR mutations **A.** Comparative protein levels and effects of 3 μM erlotinib on EGFR steady state levels in HCC827 (delE746-A750), NCI-H3255 (L858R) and NCI-H1975 (L858R/T790M) cells are shown. Cells were treated with 3 μM erlotinib, and 6 h post treatment cell lysates were prepared and subjected to immunoblotting using the indicated antibodies. Relative band intensities were calculated using Image J software considering DMSO-treated control EGFR level as 1. **B.** HCC827 and NCI-H1975 cells were treated with different (0, 0.3, 1, 3, 10 and 30 μM) concentrations of erlotinib, and 30 min post treatment cell lysates were prepared and subjected to immunoblotting using the indicated antibodies. **C.** HCC827 and NCI-H1975 cells were first treated with 3 μM erlotinib for 30 min followed by 100 ng/ml EGF as shown for the indicated time periods. Cell lysates were prepared and subjected to immunoblotting using the indicated antibodies.

**Figure 3:**
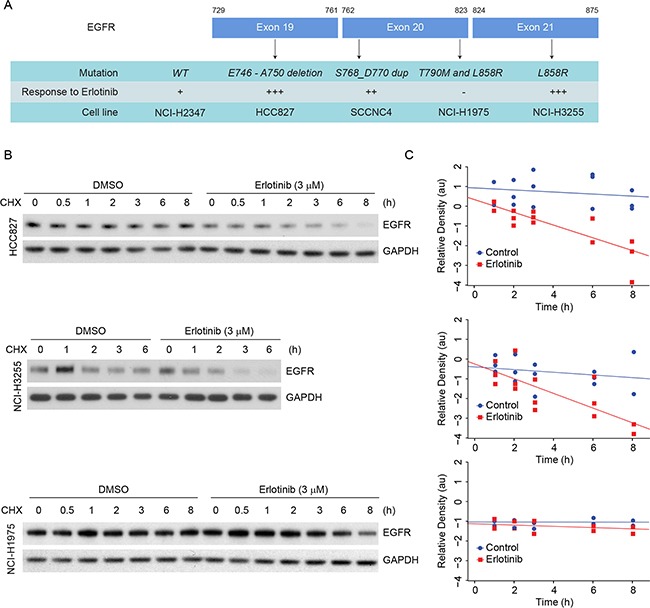
Erlotinib treatment induces faster degradation of L858R and delE746-A750 EGFR proteins in NSCLC cells **A.** List of cell lines, respective mutations and their sensitivities to erlotinib are summarized. **B.** HCC827 (delE746-A750), NCI-H3255 (L858R) and NCI-H1975 (L858R/T790M) cells harboring the above mentioned EGFR mutations were either treated with DMSO or with 3 μM erlotinib for 12 h. Following treatment, cells were treated with 50 μg/ml of cycloheximide (CHX), and cell lysates were prepared at the indicated time points. Immunoblotting analyses were performed using the indicated antibodies. **C.** Band intensities (arbitrary units, a.u.) were measured using Image J software, and each graph represents mean ± SEM from three independent experiments for an individual cell line.

To confirm the observations made in the ectopic CHO model, we selected cell lines that contain either erlotinib sensitive or resistant EGFR mutants frequently observed in patients (Figure 3A). NCI-H2347, NCI-H3255, HCC827, HCC-NC4, and NCI-H1975 endogenously expressing WT, L858R, delE746-A750, S768_D770 duplication, and L858R/T790M mutants, respectively. NCI-H3255 and HCC827 cells are sensitive to erlotinib treatment whereas, NCI-H1975 cells are resistant [25, 26]. As we observed that L858R protein is more labile than L858R/T790M protein in the ectopic system, we wished to determine whether erlotinib could induce more rapid degradation of L858R and delE746-A750 proteins compared to L858R/T790M in lung cancer cells. We first noted that the basal level of EGFR expression was substantially different among these cell lines (Figure 2A). Based on densitometry analyses, HCC827, and NCI-H3255 cells expressed about 2 and 4 times higher levels of EGFR compared to NCI-H1975 cells, respectively. In spite of such EGFR overexpression, TKI sensitive cells showed about 50% reduction in EGFR levels within 6 h of erlotinib treatment. As hypothesized, we found little change in EGFR protein levels in TKI resistant NCI-H1975 cells (Figure 2A), whereas, WT EGFR carrying NCI-H2347 cells showed an intermediate response to erlotinib-induced EGFR downregulation (Supplementary Figure S1C). As we observed differential pharmacodynamic changes in EGFR protein in TKI sensitive as compared to resistant cell lines, we further assessed if this relates to inactivation of EGFR phosphorylation. We noted that, at the basal level, TKI sensitive HCC827 cells showed about 5 fold more pEGFR when compared to TKI resistant NCI-H1975 cells. As previously noted [27] we also found that erlotinib treatment at sub-micromolar concentrations blocked EGFR and downstream ERK phosphorylation within 30 minutes in HCC827 cells (Figure 2B, left panel). The effect of erlotinib on inhibition of EGFR and ERK phosphorylation in TKI resistant NCI-H1975 cells was observed to a lesser extent (Figure 2B, right panel). Taken together, these data indicate that other than inhibition of EGFR phosphorylation, erlotinib exerts differential effects on EGFR protein stability in cells that harbor TKI sensitive or resistant EGFR mutations.

Previous studies that have investigated the (lack of) effect of erlotinib on EGFR phosphorylation in T790M mutant EGFR were performed in the presence of a high concentration of EGF (100 ng/ml) [28]. To determine if erlotinib blocks EGF-induced phosphorylation in TKI resistant cells, cells were treated with erlotinib in the presence of EGF. As expected, EGF treatment increased EGFR phosphorylation (pY1173) in a time-dependent manner in both HCC827 and NCI-H1975 cells (Figure 2C). In spite high concentration of EGF treatment, erlotinib inhibited ligand-induced EGFR and ERK phosphorylation in both the cell lines when compared to the EGF controls.

### Erlotinib-induced down-regulation of L858R and delE746-A750 mutant EGFR is due to protein degradation

To determine if erlotinib-induced mutant EGFR down-regulation were due to protein degradation, cells were treated with either DMSO or 3 μM erlotinib. Twelve hours post treatment cells were treated with 50 μg/ml of cycloheximide (CHX), and samples were collected at different time points. Samples collected just at the time of CHX addition were considered as the 0 h time point. We found that 3 μM erlotinib treatment reduced the L858R and delE746-A750 protein half-lives to about 1.66 ± 0.39 and 1.92 ± 0.39 h, respectively, as compared to >8 h in case of DMSO-treated control cells (Figure 3A, 3B, upper and middle panels). A much lower concentration of erlotinib (300 nM) remained effective in reducing the half-life of EGFR compared to controls (Supplementary Figure 1D). In contrast, in NCI-H1975 cells carrying L858R/T790M EGFR mutations, protein half-life remain unchanged (>8 h) both in the presence and absence of erlotinib (Figure 3A, 3B, lower panels). Such data indicate that erlotinib causes EGFR degradation only in the cases of L858R and delE746-A750 mutants, whereas, L858R/T790M mutants are stable, which correlates with cell death and the clinical outcome.

**Figure 4:**
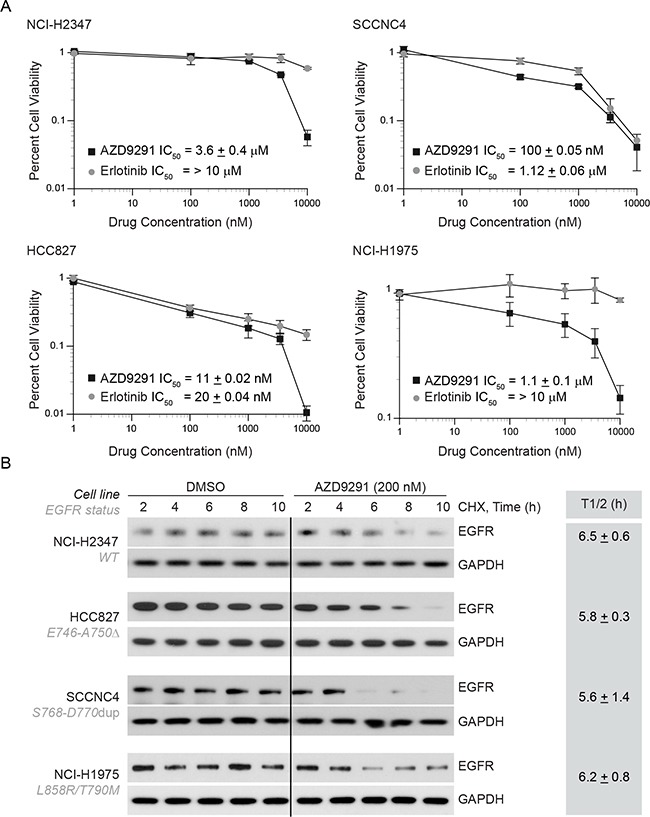
AZD9291 treatment is effective in erlotinib-resistant cells and induces EGFR degradation independent of kinase mutations **A.** NCI-H2347 (WT), SCCNC4 (S768-D770 dup), HCC827 (delE746-A750), and NCI-H1975 (L858R/T790M) cells harboring the above mentioned EGFR mutations were either treated with DMSO, erlotinib or AZD9291. Four days after treatment cellular viability was assessed using MTT assay and results were plotted relative to vehicle control. **B.** To assess the effect of AZD9291 on EGFR half-life, cell-lines described in Figure 4A were treated as described in Figure 3, and EGFR levels were measured. The mean half-life ± SEM from three independent experiments for each cell line is shown in the box.

Third-generation EGFR TKI such as AZD9291 is known to inhibit EGFR phosphorylation independent to EGFR kinase mutations and appears to be effective for patients with T790M-EGFR tumors. We hypothesized that AZD9291 treatment would be more effective in erlotinib-resistant NCI-H1975 cells and that it would induce EGFR degradation in these cells. To test this idea, we selected 4 cell lines that are driven by either WT- or mutant-EGFR (Figure 3A) and compared the cellular viability in response to either erlotinib or AZD9291 using an MTT assay. As shown in the Figure 4A, we observed that both erlotinib and AZD9291 were effective in the low-nanomolar range in reducing percent cell viability of HCC827 cells. Against, cells that are either driven by WT-EGFR or by exon 20 insertion, AZD9291 seems more effective compared to erlotinib. In erlotinib-resistant NCI-H1975 cells, AZD9291 was effective in reducing cellular viability where erlotinib was completely ineffective. We next determined if this difference in cellular response also correlated with EGFR degradation in these cell lines, we performed similar experiments to those described in Figure 3 and determined the influence of AZD9291 on the half-life of EGFR (Figure 4B and gray box). We found that AZD9291 reduced the half-life of EGFR in all 4 cell lines including in erlotinib-resistant NCI-H1975 cells. These data suggest that response to TKI correlates with its ability to reduce EGFR protein stability.

**Figure 5:**
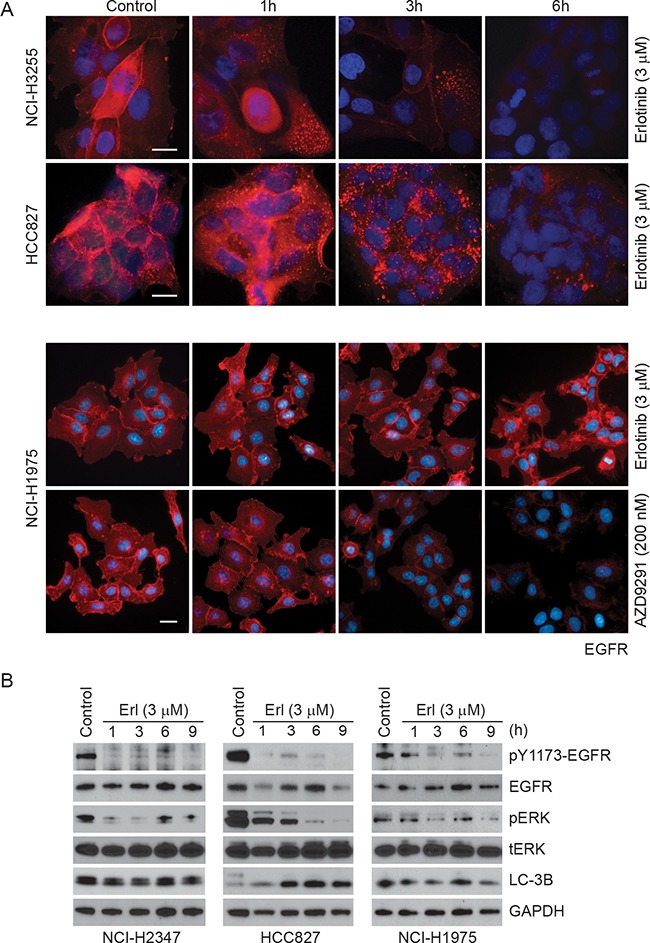
Time-dependent changes in EGFR immunofluorescence upon erlotinib/AZD9291 treatments in NSCLC cells **A.** HCC827 (delE746-A750), NCI-H3255 (L858R) and NCI-H1975 (L858R/T790M) cells were treated either with DMSO (control) or with 3 μM erlotinib/200 nM AZD9291 for the indicated time points. Cells were fixed and stained with EGFR antibody (red) and DAPI (blue, DNA dye) as described in the Materials and Methods. Representative images showing decreased EGFR immunofluorescence in erlotinib-sensitive HCC827 and NCI-H3255 cells. AZD9291 also reduced EGFR immunofluorescence even in NCI-H1975 cells. Scale bars, 10 μm. **B.** NCI-H2347, HCC827, and NCI-H1975 cells were either treated with DMSO (control) or with 3 μM erlotinib for the indicated time periods. Cell lysates were subjected to immunoblotting using the indicated antibodies.

To further investigate if TKI treatment affects EGFR localization, we performed immunofluorescence studies in NCI-H3255, HCC827 and NCI-H1975 cells (Figure 5A). These studies suggest that in erlotinib responsive HCC827 and NCI-H3255 cells EGFR is internalized within an hour of drug exposure followed by its disappearance between 3-6 h post-treatment. In contrast, NCI-H1975 cells carrying L858R/T790M mutant EGFR showed minimal change in EGFR localization or its expression. In contrast, NCI-H1975 cells, carrying L858R/T790M mutant EGFR, showed minimal change in EGFR localization or its expression in response to erlotinib. As discussed above in the Figure 4B, we found that AZD9291 was effective in reducing EGFR expression in these cells. These results are consistent with our findings described in the Figure 3 and 4, suggesting differential effects of erlotinib on protein stability of EGFR mutants. Furthermore, we noted increased nuclear localization of EGFR primarily in NCI-H1975 cells. Previously, nuclear EGFR has been associated with faster disease progression, poor survival, enhanced resistance both to radio/chemo and anti-EGFR therapies including gefitinib and cetuximab [29-32]. As loss of EGFR is known to induce cell death primarily via the autophagic mode, we treated different lung cancer cells (NCI-H2347, HCC827, and NCI-H1975 carrying wild-type, delE746-A750 and L858R/T790M mutant EGFR respectively) with erlotinib and tested the mode of cell death using markers of autophagic (LC-3B) cell death. As shown in Figure 5B, erlotinib-sensitive HCC827 cells showed a time-dependent increase in both autophagic and apoptotic markers, whereas, the other two cell lines (NCI-H2347 and NCI-H1975) showed minimal change in any cell death marker, which was correlated with a lack of sensitivity to erlotinib.

To address the possibility that erlotinib treatment alters EGFR at the transcript level, we performed quantitative RT-PCR (qRT-PCR) from RNA isolated from the three different cell lines treated with either DMSO or erlotinib for different time periods. As shown in Supplementary Figure S3, no major differences were noted at the EGFR mRNA levels in all the cell lines tested. We conclude from these data that L858R and delE746-A750 mutants are more vulnerable to erlotinib-induced degradation compared to the L858R/T790M mutant and that this difference is not due to changes in the EGFR transcription.

### Erlotinib induces polyubiquitination-mediated proteasomal degradation

**Figure 6:**
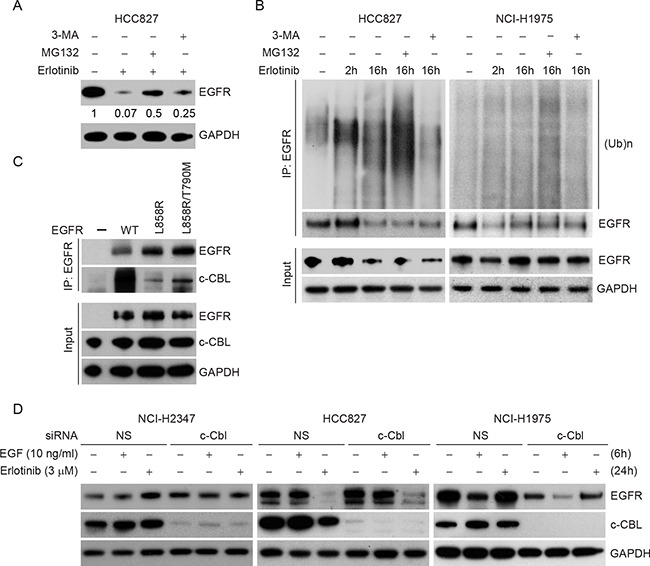
Erlotinib induces polyubiquitination-mediated proteasomal degradation in HCC827 cells **A.** HCC827 cells were either treated with DMSO or with 3 μM erlotinib for 12 h followed by treatment either with 2 μM proteasomal inhibitor, MG132 or with 5 mM lysosomal inhibitor, 3-methyladenine (3-MA) for the last four 4 h in the indicated lanes. Cell lysates were prepared and subjected to immunoblotting using the indicated antibodies. **B.** HCC827 cells (left panel) and NCI-H1975 cells (right panel) were either treated with DMSO (lane 1), with 3 μM erlotinib for 2 h (lane 2), for 16 h (lane 3) or in the presence of erlotinib for the first 12 h followed by either MG132 (lane 4) or 3-MA (lane 5) for the last four 4 h as described in Figure 5A. Cell lysates were then subjected to immunoprecipitation using EGFR antibody followed by immunoblotting using the indicated antibodies. We experienced technical difficulties in scaling NCI-H3255 cells, as after several passages these cells underwent senescence, therefore, we could not perform immunoprecipitation studies which require large numbers of cells. **C.** 500 μg of total cell lysates from CHO cells overexpressing either WT, L858R/T790M or L858R mutant EGFR were subjected to immunoprecipitation using anti-EGFR antibody and immunoblotted using the indicated antibodies. 10 μg of total cell lysates were used as input. **D.** NCI-H2347, Hcc827 and NCI-H1975 cells were transfected either with nonspecific control or c-CBL siRNA, and 24 h post-transfection cells were either left untreated or treated either with EGF (10 ng/ml for 6 h) or erlotinib (3 μM for 24 h). Cell lysates were prepared and subjected to immunoblotting using the indicated antibodies.

As erlotinib induced degradation of TKI sensitive EGFR mutant proteins, we investigated the mechanism of erlotinib-induced EGFR decay. EGFR is known to undergo degradation in response to various stimuli including EGF or chemotherapeutic agents such as cisplatin. However, the mode of degradation appears to be different; EGF mainly induces lysosomal degradation whereas cisplatin causes proteasomal degradation. To evaluate which pathway of degradation is activated by erlotinib in TKI sensitive lung cancer cells, HCC827 cells were treated with erlotinib (3 μM) for 12 h and then exposed to MG132, to inhibit proteasomal activity, or to 3-methyladenine (3-MA to block lysosomal function, followed by immunoblotting to assess rescue of EGFR protein levels. Erlotinib caused a significant decrease in EGFR protein level within 12 h of drug treatment (∼90% reduction, lane 2) (Figure 6A). Interestingly, inhibition of proteasomal activity by MG132, partially rescued (∼50% reduction) erlotinib-induced EGFR degradation (lane 3). Similarly treatment with a lysosomal inhibitor (3-MA) also rescued (∼75% reduction, lane 4), suggesting the involvement of both pathways.

As the inhibition of the proteasomal pathway significantly rescued TKI-sensitive EGFR degradation induced by erlotinib, we hypothesized that erlotinib induces degradation via polyubiquitination of EGFR. To test this idea, cells were treated with erlotinib in the presence or absence of either MG132 or 3-MA for the indicated time periods. EGFR was immunoprecipitated using EGFR-specific antibody followed by immunoblotting using ubiquitin antibody. As shown in the Figure 6B (HCC827 cells, left panel), an increase in polyubiquitinated species of EGFR was noted in the presence of MG132. Similar studies using erlotinib resistant NCI-H1975 cells resulted in no change in EGFR ubiquitination and steady-state levels (Figure 6B, right panel). Taken together, these data indicate that erlotinib specifically induces a polyubiquitination-mediated proteasomal/lysosomal degradation of EGFR in TKI sensitive cells but does not affect EGFR levels in TKI resistant cells.

Prior studies have suggested that EGF-induced EGFR degradation depends on c-CBL. To address c-CBL involvement in differential sensitivity to erlotinib with respect to EGFR mutation and degradation, we first analyzed c-CBL binding with WT, L858R, and L858R/T790M EGFR mutants. Interestingly, L858R EGFR, the most sensitive mutant to erlotinib mediated degradation, was the least associated with c-CBL (Figure 6C). In additional experiments, c-CBL knockdown in NCI-H2347 and NCI-H1975 cell lines failed to show either EGFR accumulation or EGF/erlotinib-induced alteration in EGFR steady-state levels (Figure 5D). In TKI-sensitive HCC827 cells, siRNA-mediated c-CBL knockdown caused only a minimal increase (∼1.5 fold) in EGFR steady state-levels, and the rate of erlotinib-induced EGFR downregulation was only minimally rescued (only about 10%) (Figure 6D). We also compared the gene expression profile of EGFR and c-CBL in a large data sets available in ‘cBioportal’. As shown in Supplementary Figure S4, no obvious inverse correlation between EGFR and c-CBL was noted in any of the reported studies. We performed further detailed analyses on samples from three specific studies on patients with glioblastoma multiforme (n=136), lung adenocarcinoma (n=230) and head and neck squamous cell carcinoma (n=279), where EGFR protein levels were compared with c-CBL transcript levels. Such analyses failed to show any obvious correlations (Supplementary Figure S5). Taken together, the data indicate that c-CBL has only a minimal role in the differential effects of erlotinib on different NSCLC cell lines.

### Erlotinib blocks EGFR phosphorylation *in vivo* in NCI-H1975 xenografts but does not affect tumor growth

**Figure 7:**
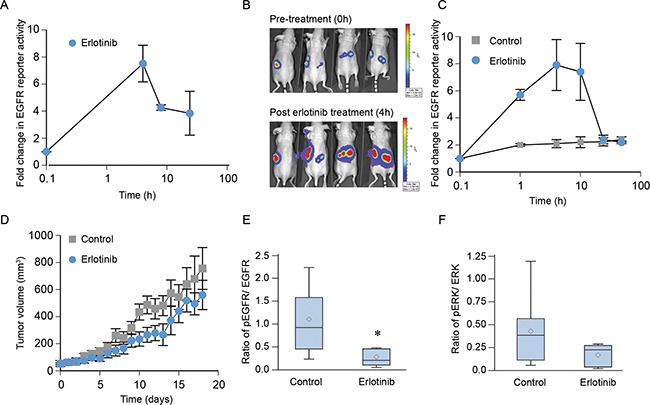
Erlotinib blocks EGFR phosphorylation *in vivo* in NCI-H1975 xenografts but not affect tumor growth **A.** Cells were treated with 3 μM erlotinib or DMSO (control) and bioluminescence activity was recorded after 10 min of treatment up to 24 h. Data were normalized to control values and plotted as mean ± SEM. **B.** *In vivo* bioluminescence activity was obtained from nude mice bearing NCI-H1975-BER flank tumors before and after treatment with erlotinib at the indicated time points. **C.** Fold change in EGFR reporter activity was calculated using the pre-treatment values as the baseline and plotted as mean ± SEM. **D.** Animals were randomized into two groups (*n*=5) and given either 100 mg/kg of erlotinib or saline as described in Materials and Methods. Tumor volume was measured daily, and tumor volume was plotted as the mean ± SEM (*p*=0.97). **E–F.** On day 18, mice were treated with 100 mg/kg erlotinib, and 4 h post-treatment animals were euthanized and their tumors were harvested. Tumor cell lysates were prepared using a standardized protocol and immunoblotted using phospho EGFR, total EGFR, phospho ERK, total ERK with GAPDH used as a loading control. The ratio of pEGFR/EGFR (panel E, *p*= 0.02) or pERK/ERK (panel F, *p*=0.14) from control (8 tumors) and erlotinib (6 tumors) treated specimens are represented using the box plot.

We used a luciferase-based reporter to investigate EGFR signaling events in real time both *in vitro* and in tumors implanted in nude mice [33]. To utilize such a system, first, we established NCI-H1975 cells stably transfected with the bioluminescence EGFR reporter (BER). In this system, *inhibition* of EGFR phosphorylation is associated with an *increase* in bioluminescence. Cells were imaged following treatments either with either DMSO or erlotinib. As shown in Figure 7A, there was a significant increase (about 7-fold) (*p* < 0.0001) in bioluminescence within 4 h of 3 μM erlotinib treatment, which gradually decreased over 24 h post-treatment (*p* = 0.0022 at 8h and *p* = 0.02 at 24 h), indicating that erlotinib inhibits EGFR phosphorylation in this cell line. Next, we confirmed the use of this real-time non-invasive method to detect an effect of erlotinib treatment on EGFR activity using a mouse xenograft model. As shown in Figure 7B and quantified in Figure 7C, imaging of animals bearing comparably sized tumors (60 mm^3^) gave a basal level bioluminescence, which increased about 8 fold within 4 h of erlotinib treatment (*p* <0.0001) and decreased back to the basal levels within 24 h post treatment (*p* =0.96). Although these *in vivo* observations suggest that the effect of erlotinib on EGFR is less durable compared to our *in vitro* model, it was consistent up to 8 hours post treatment. The effect of erlotinib treatment on tumor growth was recorded daily for 18 days. In spite of inhibition of EGFR phosphorylation, weekly administration of erlotinib failed to delay tumor growth (Figure 7D) (*p* = 0.97), which is consistent with the previous reports. We further determined the effect of erlotinib treatment on inhibition of phospho EGFR and phospho ERK using immunoblotting (Figure 7E and 7F). As shown in Figure 7E, the ratio of pEGFR/EGFR was lower in erlotinib-treated animals (*p* = 0.02). However, the change in ERK phosphorylation was not significantly different in the erlotinib-treated group (*p* = 0.14). Taken together, these data show that although erlotinib blocks EGFR phosphorylation, it failed to cause EGFR degradation and had no effect on tumor growth in T790M-driven NCI-H1975 tumors.

## DISCUSSION

From this study, we propose an additional mechanism of differential responsiveness of TKI sensitive (L858R, delE746-A750) and resistant (T790M) EGFR mutants to erlotinib treatment. Our data suggest a differential effect of erlotinib on destabilizing various EGFR mutants which, along with differential ATP affinity and drug binding of EGFR mutants, is responsible for differential therapeutic outcomes observed in subsets of NSCLC patients. Based on an ectopic EGFR expression model, cancer cell lines expressing various EGFR mutants, and a tumor xenograft model, we found that erlotinib inhibits EGFR phosphorylation across the models, but protein degradation is induced only in the case of erlotinib responsive cells, which correlates with the response. Our study adds to the potential mechanisms by which resistance to TKIs can develop and is consistent with an earlier study with PC9 cells that contained the delE746-A750 mutant EGFR. Treatment of these cells with gefitinib caused rapid internalization of surface EGFR, which was not the case in TKI resistant cell lines that contained wild-type EGFR [34]. In contrast, here we show that ligand-induced degradation of EGFR is independent of kinase mutations, as both the sensitive and the resistant EGFR mutants show a similar rate of EGFR decay upon stimulation with EGF. Our data also indicate that continuous treatment with erlotinib blocks EGFR phosphorylation and down-stream signaling in both TKI-sensitive and resistant cells, but EGFR is degraded only in the erlotinib responsive cell lines. Erlotinib induced EGFR degradation appears to be c-CBL-independent. These are novel observations and are in contrast to the notion that suggests that erlotinib treatment blocks EGFR and c-CBL binding in cells that express TKI resistant mutant EGFR, thus inhibiting EGFR degradation resulting in TKI resistance.

Our findings are consistent with the idea that although kinase activity is important for cell growth and cell division, the physical presence of the protein has functions beyond kinase activity [35]. For example, EGFR is known to protect cells from autophagy by a kinase-independent mechanism [36]. In glioblastoma, EGFRvIII is known to sequester the proapoptotic protein PUMA in a kinase-independent manner which promotes drug resistance [37]. In addition, EGFR knockout is embryonically lethal in mice [38], whereas, transgenic mice expressing a kinase-dead form of EGFR are viable with minimal defects [39]. Similarly, erlotinib treatment inhibits phosphorylation of EGFR and its down-stream effector molecule ERK in cells collected from the oral cavity of patients or normal lung fibroblasts that contain wild-type EGFR without induction of substantial cell-death [40]. These results suggest that EGFR kinase activity is important for tumorigenesis, but its physical presence might be enough to promote cell survival, likely by forming hetero- or homo- dimer formation with other family members [41]. These interactions are known to facilitate down-stream signaling through molecules such as AKT which has been implicated in resistance to gefitinib or erlotinib treatment [34, 41, 42]. Clinical findings consistent with this idea come from a phase I clinical trial in which head and neck squamous cell carcinoma (HNSCC) patients were undergoing combination treatment with the anti-EGFR antibody cetuximab with increasing doses of the proteasomal inhibitor, bortezomib. That study reported faster disease progression compared to the historic control, and molecular analysis of tumor specimens revealed stabilization of EGFR by this combination as a potential mechanism of tumor progression [17].

The mechanism by which the T790M mutation becomes dominant within a tumor is of significant interest. Earlier studies indicated that only a small percentage of NSCLC patients harbor T790M-EGFR mutations, and a higher frequency is observed only in patients treated with TKI, which coincides with acquired resistance. This observation led to the thought that T790M is an acquired mutation, which causes resistance to TKI treatment [7, 26]. Recent studies propose a condition under which this mutation could be acquired [43]. Alternatively, studies using sensitive technologies such as Matrix-assisted laser desorption/ionization (MALDI) Time of Flight-Mass Spectrometry (TOF-MS) have suggested that T790M-EGFR may be a *de novo* mutation, and its presence correlates with the duration of response to TKI's [44]. These findings suggest that treatment with TKI might not be causing the induction of a new point mutation (T790M), but cells that contain this mutation are selected for during treatment with EGFR TKIs.

Furthermore, computational and structural analyses have also confirmed that TKIs can efficiently bind with either inactive or active conformations of EGFR [45]. However, Gibbs free energy landscape analysis predicts that WT-EGFR is relatively more stable compared to highly active L858R-EGFR protein, which is thermodynamically labile. Further structural and thermodynamic analysis of EGFR shows that EGFR prefers the inactive conformation to gain thermodynamic stability [46]. A previous computational study predicted that T790M or L858R -EGFR mutants are more stable in the active state [47]. Our findings are in agreement with this analysis, which suggests that L858R-EGFR is more active compared to both WT- or T790M-EGFR, that show reduced tyrosine phosphorylation when compared to TKI sensitive L858R-EGFR. The exact relationship between EGFR kinase thermodynamic stability and overall protein stability with respect to TKI mutants needs further investigation.

Several ubiquitin ligases (E3) have been shown to regulate EGFR protein stability [48-52]. Amongst those c-CBL is most studied E3 controlling EGFR protein levels. In order to determine the role of c-CBL in differential degradation of EGFR mutants, we assessed EGFR and c-CBL binding, the effect of c-CBL knockdown on steady state levels of EGFR and c-CBL's effect on EGF and erlotinib-induced EGFR degradation (Figure 5). We have noted that ligand-induced EGFR downregulation may be a c-CBL independent process, and our data indicate that binding of TKI sensitive L858R EGFR with c-CBL is, in fact, minor when compared to WT and L858R/T790M mutant. Overall, our data indicate a minimal role of c-CBL in regulating EGFR protein stability. This is consistent with the recent findings from the Band laboratory that Cbl is dispensable for internalization and degradation of EGFR in mouse embryonic fibroblasts (MEFs) from c-CBL knockout mice [53]. In addition, we found no correlation between EGFR and c-CBL mRNA expression across different cancer types (Supplementary Figure S4) and between EGFR protein expression and c-CBL mRNA levels in cancer known to be driven by EGFR (Supplementary Figure S5).

EGFR is known to regulate tumor progression and the autophagic process [54], but the role of TKI sensitivities/resistant mutation in autophagic death is not clear [36, 55, 56]. For instance, TKI resistant PC9 cells undergo autophagy upon knock-down of T790M-EGFR using si-RNA [57]. A recent study found that TKI sensitive EGFR mutant cells show activation of the autophagic process upon treatment with TKI. Inhibition of autophagy conferred resistance to TKI [56]. We have also reported that loss of EGFR protein upon knock-down of the E3 ligase *SMURF2*, induced autophagy and sensitized tumor cells [58]. Overall, these studies suggest a potential role of autophagic death in TKI sensitive EGFR mutant tumors. In this study (Figure 5B, 5C) we have confirmed the induction of both autophagic and apoptotic cell death upon treatment of TKI-sensitive HCC827 cells with erlotinib. In contrast, TKI resistant NCI-H1975 (L858R/T790M mutant EGFR) cells showed minimal alteration of LC-3B with no detectable PARP cleavage (not shown). These responses correlate well with erlotinib effects on EGFR protein stability.

Currently, the treatment options for patients with TKI resistant mutations in EGFR including T790M are limited. However, recent development of 3^rd^ generation of TKI's (such as AZD9291) has shown promising results in early clinical trials. What is the precise difference in the mechanisms of action between erlotinib and AZD9291 is not clear at this time. However, we also observed that AZD9291 is effective in reducing cell viability of erlotinib-resistant cells, and that it affects EGFR protein stability where erlotinib is completely ineffective. Our findings suggest that resistance to TKIs may partly be attributed to increased protein stability of T790M mutant EGFR. This led us to hypothesize that the molecular regulator(s), which may be cooperating with EGFR via blocking the mutant protein from undergoing proteasomal degradation, may be critical therapeutic targets in overcoming resistance. In our previous studies, we have identified two such factors, chaperone heat shock protein 90 (HSP90) [18, 19] and Smad ubiquitination regulatory factor 2 (SMURF2) [58], which provide increased EGFR protein stability. In the light of the current observation, it would be interesting to decipher whether HSP90 and/or SMURF2 are differentially involved in providing protein stability to the EGFR L858R/T790M mutant. Furthermore, it remains to be determined if these factors indeed play any role either in response to TKI's or in the development of resistance.

## MATERIALS AND METHODS

### Reagents

Anti-EGFR (sc-03) and anti-ubiquitin (P4D1) antibodies were acquired from Santa Cruz Biotechnology (Santa Cruz, CA). Antibodies for phosphotyrosine 1173 EGFR, phospho ERK, total ERK, c-CBL, LC-3B and GAPDH were purchased from Cell Signaling (Danvers, MA), whereas another EGFR antibody (31G7) and Lipofectamine were purchased from Invitrogen (Grand Island, NY). Cycloheximide (CHX) was obtained from Sigma-Aldrich (St Louis, MO), and erlotinib was obtained from Genentech Inc. (San Francisco, CA). Proteasomal inhibitor MG132 and lysosomal inhibitor 3-methyladenine (3-MA) were purchased from Calbiochem (La Jolla, CA) and Sigma (St Louis, MO), respectively. Non-specific and *c-CBL* small interfering RNA (siRNA) were purchased from Santa Cruz Biotechnology (Santa Cruz, CA).

### Cell culture

EGFR-null CHO cells and human lung adenocarcinoma HCC827 and NCI-H2347 cells were purchased from the American Type Culture Collection (ATCC). The human lung adenocarcinoma cell line NCI-H3255 was provided by the National Cancer Institute, and the lung cancer cell line NCI-H1975 was kindly provided by Dr. J. A. Engelman (Massachusetts General Hospital, Boston). SCCNC4 cell line was a gift from Mario Hermsen (Instituto Universitario de Oncología, Spain). NCI-H3255 cells were grown in RPMI-1640 medium supplemented with insulin (20 μg/ml), transferrin (10 μg/ml), sodium selenite (25 nM), hydrocortisone (50 nM), EGF (1 ng/ml), ethanolamine (10 μM), phosphorylethanolamine (10 μM), triiodothyronine (100 pM), bovine serum albumin (2 mg/ml), HEPES (10 mM), sodium pyruvate (0.5 mM) and L-glutamine (2 mM). All other cells were grown in RPMI 1640 medium supplemented with 10% fetal bovine serum. For all *in vitro* experiments, cells were released from flasks using PBS containing 0.25% trypsin and 0.2 mM EDTA, and cells were plated onto culture dishes one day prior to any treatment. All the cell lines are routinely tested for pathogen and genotyped to confirm their authenticity.

#### MTT assay

The cell proliferation assay was performed using MTT kit (Roche product # 11465007001) and assays were performed according to the manufacturer's protocol. In brief, 3000 cells in 100 μl of complete medium were plated per well in a 96-well plate 24 h prior to drug treatment. Cells were then either treated with vehicle (DMSO) or serial dilutions of TKIs (either erlotinib or AZD9291). Four days following treatments MTT labelling reagent was added, and cells were allowed to form formazan crystals for 2 h. Following, 100 μl of solubilizing agent (10% SDS in 0.01M HCl) was added per well, and plates were incubated at 37° C overnight. The optical density (OD) of the solubilized formazan was spectrophotometrically quantified using a plate reader at 570 nm with a reference at 650 nm. Data represent the mean (± standard error, SE) performed in quadruplicate, and percent cell viabilities are plotted in semi-logarithmic scale relative to DMSO treated control.

### Transfection and protein analyses

CHO cells were transfected using Lipofectamine reagent according to the manufacturer's protocol. For siRNA Lipofectamine RNA_i_max (Invitrogen) was used as described previously [58]. Immunoblot analysis and immunoprecipitation techniques were performed as described previously [58] using lysis buffer consisting of 50 mM HEPES-KOH (pH 7.5), 150 mM NaCl, 1.3 mM CaCl_2_, 1 mM DTT, 10 mM β-glycerophosphate, 1 mM NaF, 0.1 mM sodium orthovanadate, 10% glycerol, 1% NP-40, and 1x protease inhibitor cocktail (Sigma; Cat. No. P8340). For cell lysate preparation, cells were washed once with ice-cold PBS followed by addition of the required amount of lysis buffer. Cells were scraped and sonicated. After sonication, particulate materials were removed by centrifugation at 13,000 rpm for 5 min at 4 °C. The soluble protein fraction was mixed with 1X Laemmli buffer and heated to 95 °C for 5 min, then applied to a 4–12% Bis-Tris precast gel (Invitrogen), and transferred onto a PVDF membrane. Membranes were incubated for 1 h at room temperature in blocking buffer consisting of 3% BSA and 1% normal goat serum in Tris-buffered saline (137 mM NaCl, 20 mM Tris-HCl (pH 7.6), 0.1% (v/v) Tween 20). Membranes were subsequently incubated overnight at 4 °C with 1 μg/ml primary antibody in blocking buffer, washed, and incubated for 1 h with horseradish peroxidase-conjugated secondary antibody (Cell Signaling). After three additional washes in Tris-buffered saline, bound antibody was detected by enhanced chemiluminescence plus reagent (GE Healthcare). For quantification of relative protein levels, immunoblot films were scanned and analyzed using ImageJ 1.44p software (National Institutes of Health, Bethesda). The relative protein levels shown represent a comparison to untreated controls. For EGFR immunoprecipitation, studies were performed as described previously [19].

### Live fluorescence microscopy and protein decay studies

CHO cells were transfected with an equal amount (3 μg) of DNA templates of WILD-TYPE, L858R or L858R/T790M mutant EGFR constructs. Twelve hours post-transfection, cells were either treated with DMSO or with 3 μM erlotinib and EGFR-YFP expression and localization were monitored at regular intervals. Fluorescence and phase-contrast microscopic images were captured using a DS-Fi1 (Nikon, Melville, NY) camera fitted on an Olympus 1X-71 microscope. For comparative analyses fluorescence images were captured keeping the exposure time constant.

For protein decay studies, EGFR expressing cells were treated 12 h post transfection with DMSO or erlotinib, and cell lysates were prepared at specific time points. Immunoblotting was carried out for EGFR and GAPDH to analyze any alteration of EGFR steady state levels with time.

### Protein half-life studies

The effect of erlotinib or AZD9291 on EGFR half-life was determined in various cell lines known to harbor different EGFR mutations (Figure 3A). A day before treatment with TKI, cells were plated at 40 percent confluency and either treated with DMSO (vehicle control) or with erlotinib (3 μM) or AZD9291 (200 nM). Twelve hours later, protein synthesis was blocked by treatment with freshly prepared cycloheximide (50 μg/ml). Cells were harvested at the indicated times post-treatment, and immunoblotting was carried out for total EGFR and GAPDH to analyze the protein half-life of EGFR. The approximate EGFR protein half-life (t_1/2_) in the absence and presence of TKI was calculated using data (mean ± SEM) from three independent experiments by plotting relative band density (arbitrary units) and time (h) on a log-linear scale.

### Immunofluorescence studies

Cells were grown in 100-mm Petri dishes on sterile glass coverslips for a day. After treatment, coverslips were removed and fixed with 10 percent phosphate buffered formalin for 20 minutes at room temperature. Cells were washed once with TBS and permeabilized with 100 percent methanol (-20° C) for 5 minutes and rehydrated with TBS for 20 minutes. Non-specific antigens were blocked (5% goat serum, 1% BSA, 0.2% Triton x100 in TBS) for an hour at room temperature. EGFR or LC-3B expression was assessed by incubation of cells with anti-EGFR (Sc-03, 1:100) or anti-LC-3B antibody (CST cat# 3868, 1:200) at 4° C overnight. The slides were then washed with TBS thrice, incubated with the fluorescence-conjugated secondary antibodies for 1 h (488 or 594 Alexa Fluor conjugated secondary antibody, 1:100 dilution), washed thrice, and prepared with a coverslip after a drop of ProLong Gold anti-fade reagent with 4′, 6-diamidino-2-phenylindole (Molecular Probes) was added to each sample. Fluorescence images were acquired using a DS-Fi1 (Nikon, Melville, NY) camera fitted on an Olympus 1X-71 microscope.

### RNA isolation and quantitation

Total cellular RNA was isolated using a Qiagen RNeasy mini kit (Qiagen, Inc, Valencia, CA) according to the manufacturer's instructions. For quantitation, RNA samples (1 μg) were reverse transcribed with random hexamers using the High-Capacity cDNA Reverse Transcription System (Applied Biosystems, Foster City, CA). Real-time polymerase chain reaction (PCR) was carried out with an ABI Prism 7700 sequence detector using Power SYBR GREEN PCR master mix (Applied Biosystems). The following human gene-specific primers were used for the PCR reaction: 1) EGFR (forward, 5′ CAGCGCTACCTTGTCATTCA 3′ and reverse, 5′ TGCACTCAGAGAGCTCAGGA 3′) and 2) GAPDH (forward, 5′ GAGTCAACGGATTTGGTCGT 3′ and reverse, 5′ TTGATTTTGGAGGGATCTCG 3′).

### Animal studies

All animal experiments were performed according to University of Michigan-approved protocols and conform to their relevant regulatory standards. A suspension of erlotinib was made in saline with 0.1% Tween-80. Athymic nude female mice (4-5 weeks old) (Harlan Laboratories) bearing NCI-H1975-EGFR reporter [bioluminescent EGFR reporter; BER [33] flank tumors were dosed at 100 mg/kg via oral gavage on days 1 and 8 for efficacy studies. Control mice were treated with vehicle. Tumor length and width were measured every day beginning on day 0 (pre-treatment). Tumor volume was calculated as follows: volume (cm^3^) = (L x W^2^)/2. When tumor volume reached 10-fold from the day treatment began (day 18), mice were re-treated with erlotinib. Four hours later they were euthanized, and tumors were harvested. The effects of erlotinib on phospho EGFR, total EGFR, phospho ERK1/2, and total ERK1/2 were analyzed by immunoblotting.

### *In vivo* bioluminescence imaging

Live-cell bioluminescence imaging was performed on the mice using the IVIS imaging system (Caliper Life Sciences, Hopinkton, MA, USA). Mice were injected with 100 μL of 40 mg/mL D-luciferin dissolved in PBS and anesthetized with 2% isoflurane. 5 minutes after luciferin injection, bioluminescence images were acquired. Imaging was performed before the first erlotinib/vehicle treatments (pre-treatment) and at 1, 4, 8, 24, and 48 hours following treatment. Fold-change in EGFR reporter activity at each time point was calculated using pretreatment values as baseline measurements.

### Statistics

A linear regression was used to model the level of EGFR protein as a function of time, and an interaction between the treatment group and time point was tested to compare the rate of protein degradation between controls and erlotinib. The half-life of EGFR protein (corresponds to 50% reduction of the EGFR) was calculated from the estimated regression function, with a standard error obtained using the delta method. A linear mixed effects model was used to estimate the tumor growth. A random effect was included in the model to consider the correlation between the two tumors within the same animal. An ANOVA model was used to compare the relative change in bioluminescence between groups at a particular time point. The difference in pEGFR/EGFR or pERK/ERK between control and erlotinib was assessed by a two-sample Wilcoxon test. Statistical significance was defined as a two-sided *P*-value <0.05. All analyses were conducted using SAS (version 9.4, SAS Institute, Cary, NC).

## SUPPLEMENTARY MATERIALS FIGURES


