# Supplementary material for: Differential protein stability of EGFR mutants determines responsiveness to tyrosine kinase inhibitors
Source: Oncotarget. 2016 Sep 6;7(42):68597–613. doi: 10.18632/oncotarget.11860 (PMC5356576; doi:10.18632/oncotarget.11860)
Supplement: Supplementary file 1 [file oncotarget-07-68597-s001.pdf]

Differential protein stability of EGFR mutants determines responsiveness to tyrosine kinase inhibitors

SUPPLEMENTARY FIGURES

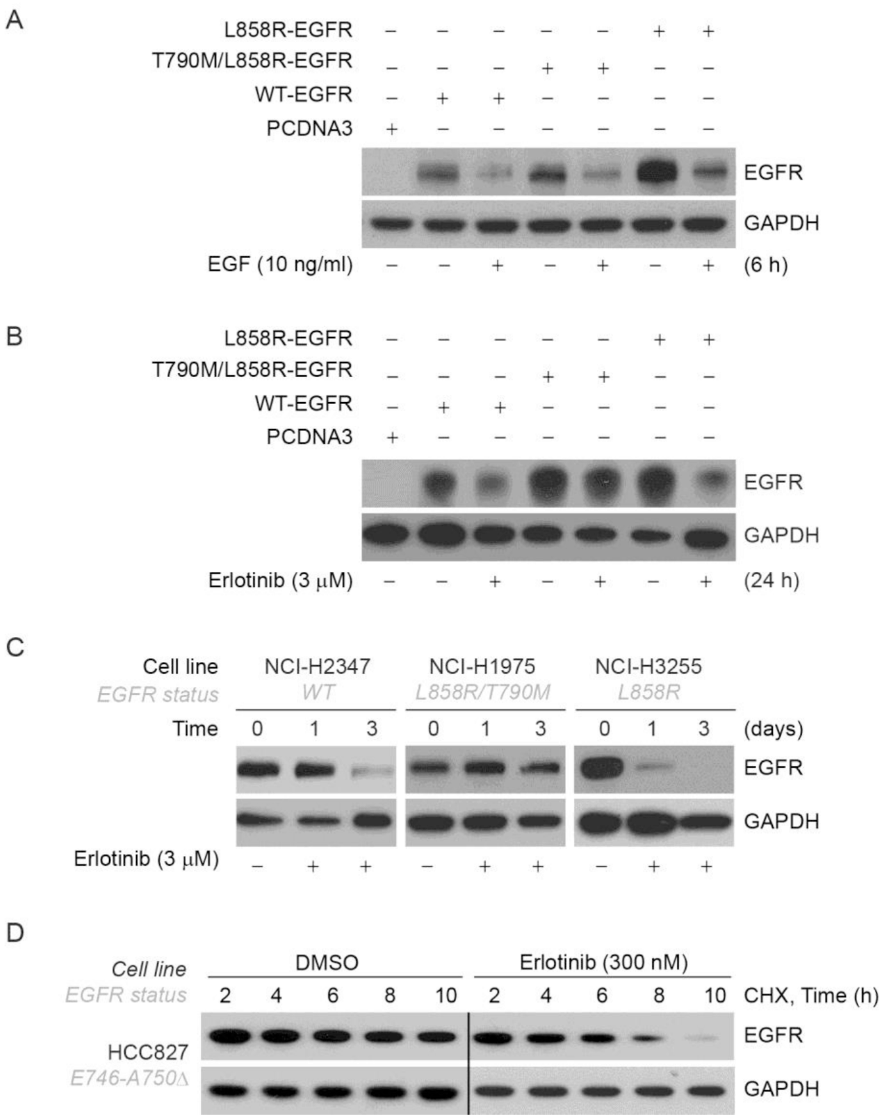

Supplementary Figure S1: Effect of EGF and erlotinib on steady state levels of different EGFR mutations. A. CHO cells overexpressing either wild-type (WT), L858R or L858R/T790M mutant EGFR were either left untreated or treated with 10 ng/ml EGF. Six hours post-treatment cell lysates were prepared, and EGFR levels were analyzed by immunoblotting with anti-EGFR antibody. GAPDH was used as a loading control. **B.** CHO cells expressing either WT or mutant EGFR (L858R, L858R/T790M) were treated with 3 μM erlotinib, and 24 h post-treatment cell lysates were prepared and subjected to immunoblotting using the indicated antibodies. **C.** NSCLC cell lines known to express erlotinib sensitive or resistant EGFR mutants were treated with 3 μM erlotinib daily. Cell lysates were prepared at the indicated time points and subjected to immunoblotting using the indicated antibodies. **D.** The effect of 300 nM erlotinib on EGFR protein stability in HCC827 cells was analyzed as described in Figure 4. Controls blots are the same blots as shown in Figure 4.

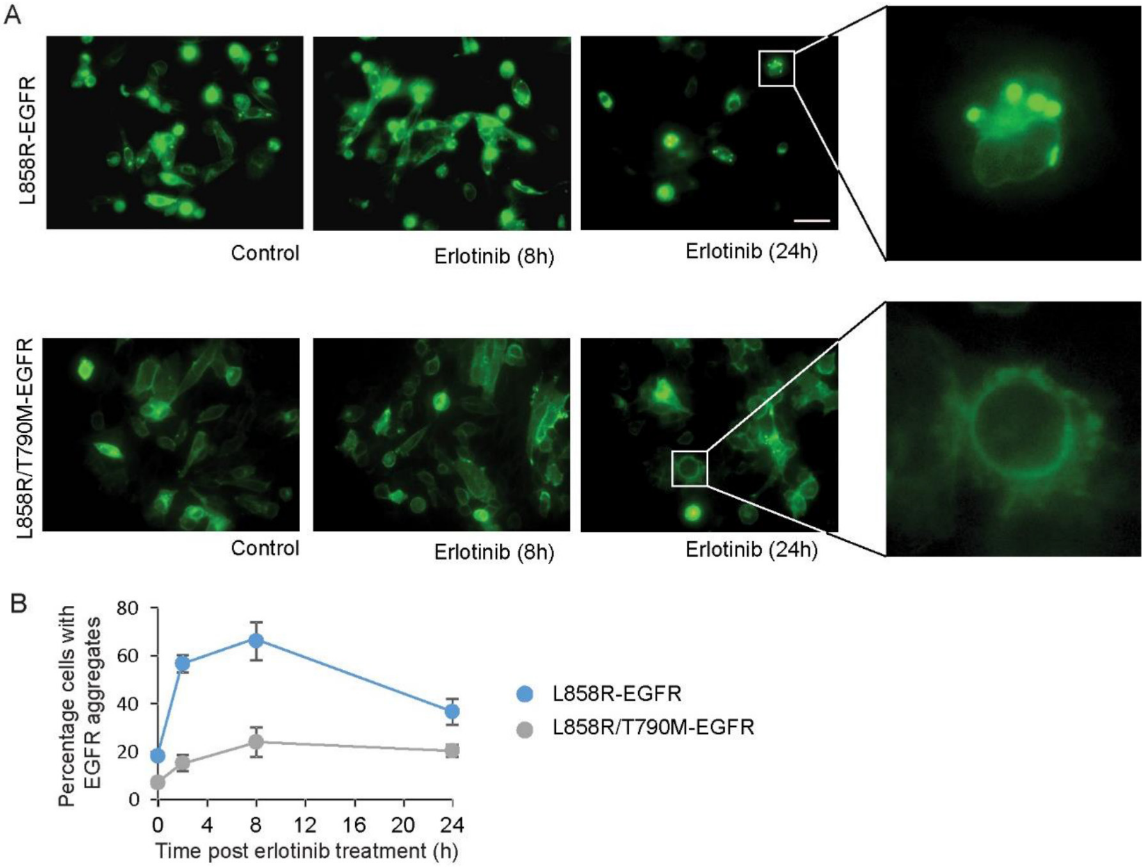

Supplementary Figure S2: Erlotinib treatment enhances L858R-YFP protein intracellular aggregation in CHO cells. **A.** Fluorescence microscopy images of transiently transfected CHO cells expressing either L858R or L858R/T790M YFP-tagged EGFR proteins. Cells were either left untreated (control) or treated with 3 µM erlotinib, and images were captured at 8 and 24 h following drug treatment. The insets show a magnified image of a cell. **B.** Based on fluorescence images, the total number of YFP-EGFR transfected cells and the number of cells showing aggregated YFP protein were counted from ten different, randomly selected fields. The percentage of cells with EGFR aggregates was calculated and plotted at the indicated times following erlotinib treatment. Error bars represent mean ± SEM. Scale bar, 10 µm.

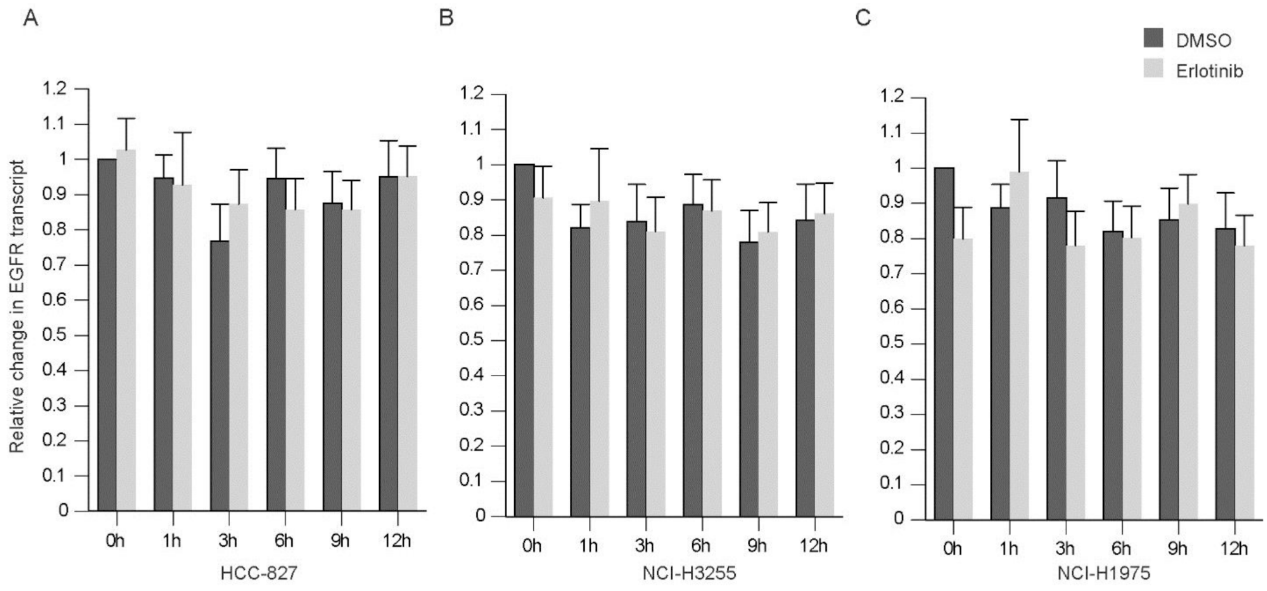

Supplementary Figure S3: Minimal alterations at the EGFR transcript level upon erlotinib treatment in NSCLC cells. HCC827 (in **A**), H3255 (in **B**) and H1975 (in **C**) cells were either treated with DMSO or with 3 μM erlotinib for the indicated time periods. Total RNA was isolated, quantified and subjected to quantitative real-time PCR (qRT-PCR) using EGFR and GAPDH specific primers as described in the materials and methods. Results represent mean ± SEM from three independent experiments for each cell line.

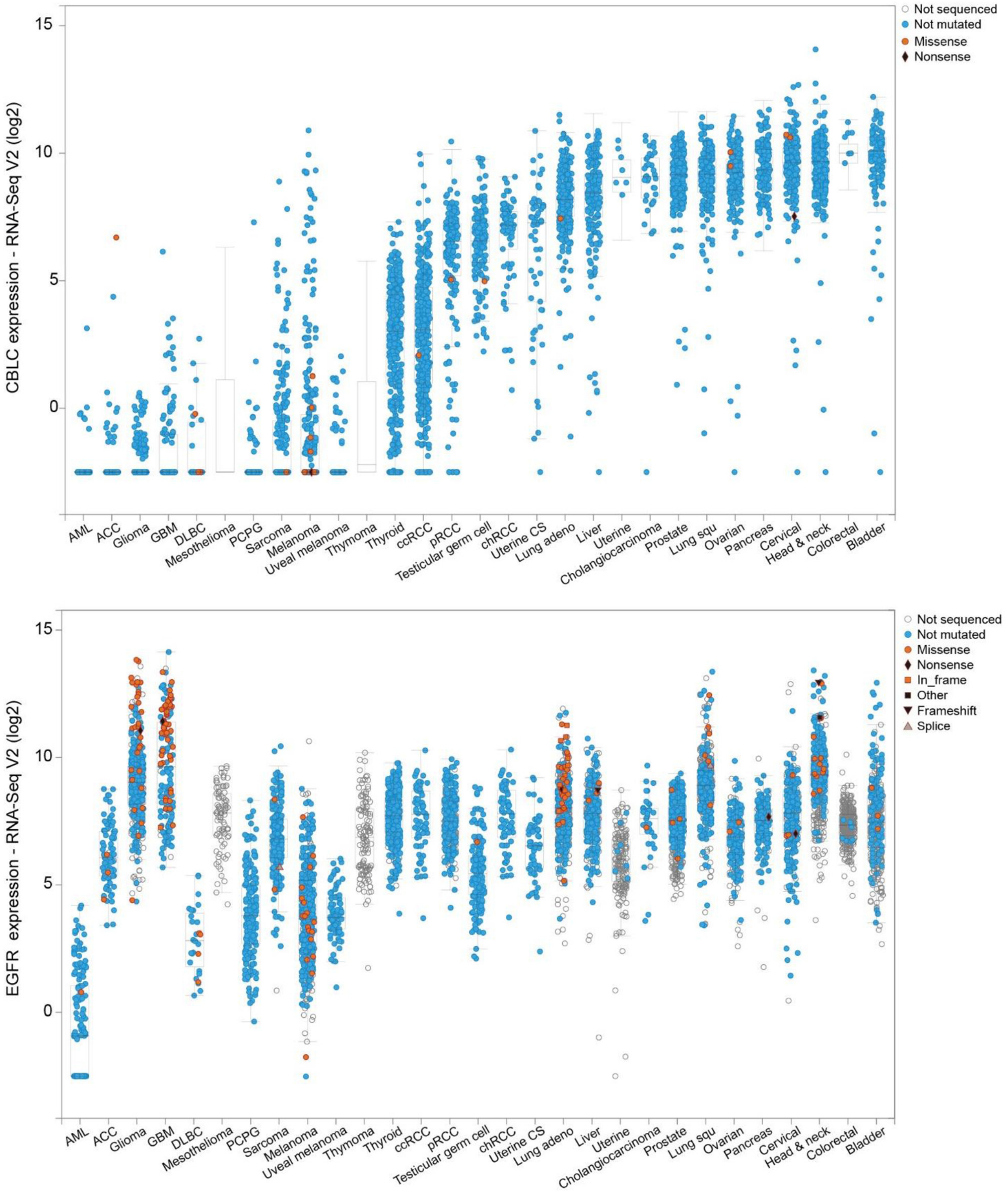

Supplementary Figure S4: Correlation between EGFR and c-CBL mRNA expression across different cancer types. Scatter plots showing a lack of relationship between EGFR and c-CBL mRNA expression across cancer types. Data are plotted from http://www.cbioportal.org/

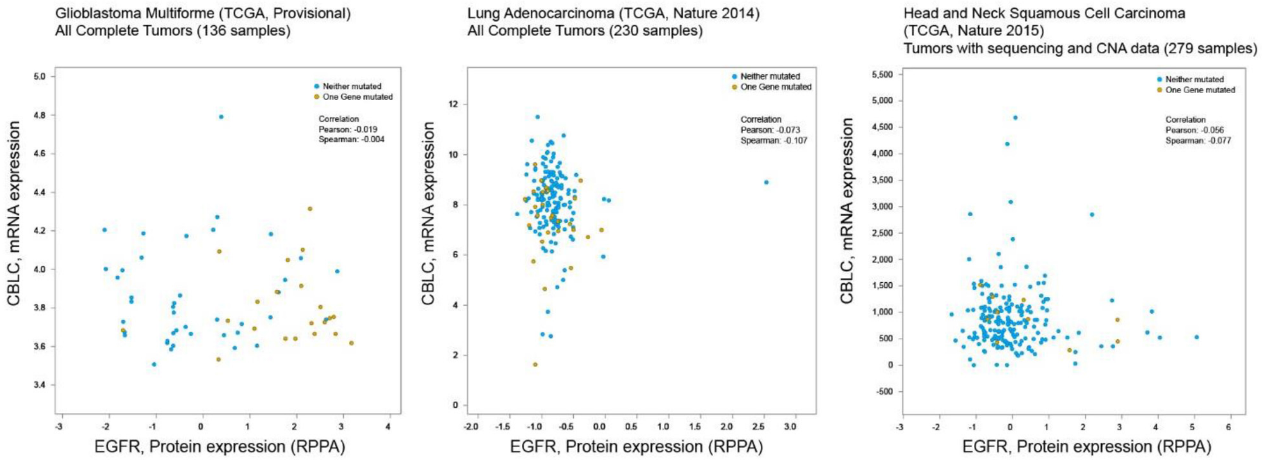

Supplementary Figure S5: Correlation between EGFR protein levels and c-CBL mRNA in selected cancer studies. Three plots showing lack of relationship between EGFR protein levels and c-CBL mRNA expression in glioblastoma multiforme, lung adenocarcinoma and head and neck squamous cell carcinoma (data-base available from http://www.cbioportal.org/).
